# A successful case of endoscopic submucosal dissection using the water pressure method for hypopharyngeal carcinoma with severe fibrosis

**DOI:** 10.1055/a-2079-2910

**Published:** 2023-05-15

**Authors:** Kurato Miyazaki, Motohiko Kato, Yusaku Takatori, Takanori Kanai, Naohisa Yahagi

**Affiliations:** 1 Division of Gastroenterology and Hepatology, Department of Internal Medicine, Keio University School of Medicine, Tokyo, Japan; 2 Division of Research and Development for Minimally Invasive Treatment, Cancer Center, Keio University School of Medicine, Tokyo, Japan

Endoscopic submucosal dissection (ESD) is a local treatment method for pharyngeal carcinoma that can preserve organs and maintain patientsʼ quality of life. However, it is challenging because of the narrow and three-dimensional structure, which makes it difficult to use a traction method like with other organs. We have previously reported the usefulness of the water pressure method (WPM) in other organs 1 2 3 4 5 . In addition to the magnified effect obtained by underwater ESD, WPM makes it easier to get under the mucosal flap and identify the submucosal edge to be dissected by using the water stream. Here, we report a successful ESD using WPM for hypopharyngeal carcinoma ( Video 1 ).

**Video 1** A successful case of endoscopic submucosal dissection using water pressure method for hypopharyngeal carcinoma with severe fibrosis.


A 62-year-old man underwent ESD for hypopharyngeal carcinoma 5 years ago, and a curative resection was achieved. At surveillance endoscopy, a new hypopharyngeal carcinoma was detected adjacent to the post-ESD scar, and we performed ESD.

We identified the lesion with iodine staining, and marking dots were placed around the lesion ( Fig. 1 ). First, we performed a mucosal incision at the distal edge to ensure the endpoint of the subepithelial dissection prior to circumferential incision. Thereafter, this case was very challenging because of the narrow space and the vertical approach to the muscular layer. In addition, fibrosis caused by the post-ESD scar was one of the difficulties in this case ( Fig. 2 ). However, WPM made it possible to visualize the edges of the layers to be dissected and distinguish between the fibrotic tissue and the muscle layer. Furthermore, the tapered hood and the magnifying effect in the underwater condition enabled us to perform precise dissection ( Fig. 3 ). En bloc resection was achieved without any adverse events ( Fig. 4 ) despite severe fibrosis ( Fig. 5 ).

**Fig. 1:**
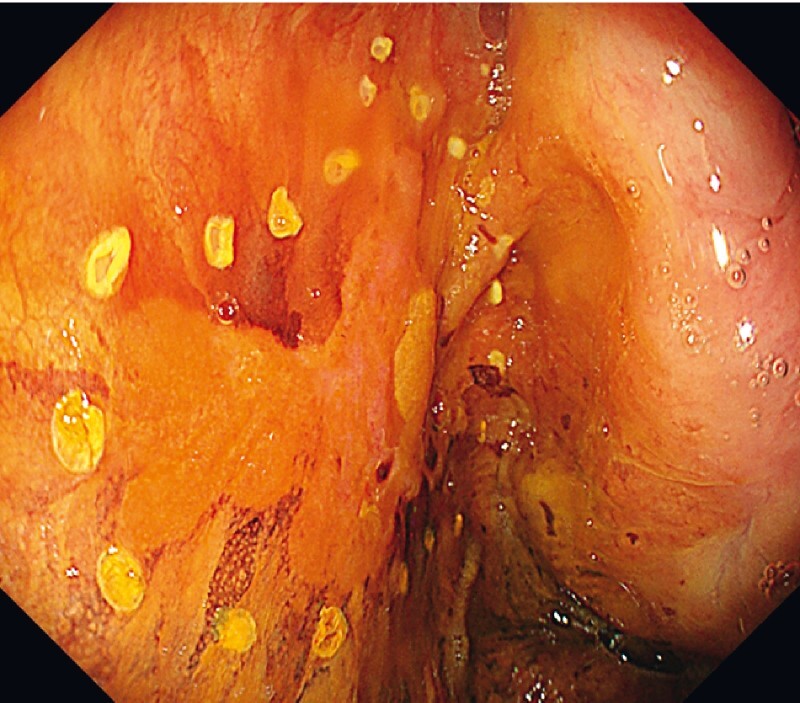
A white light image of the lesion with iodine staining. After iodine staining, marking dots were placed around the lesion.

**Fig. 2:**
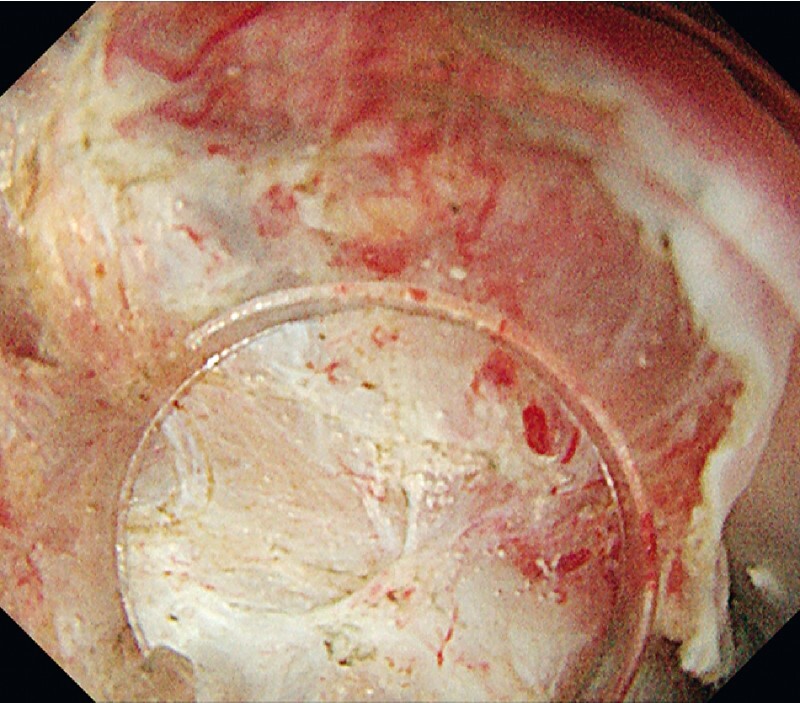
A white light image of severe fibrosis. Severely fibrotic tissue due to post-endoscopic submucosal dissection (ESD) scar spread adjacent to the lesion.

**Fig. 3:**
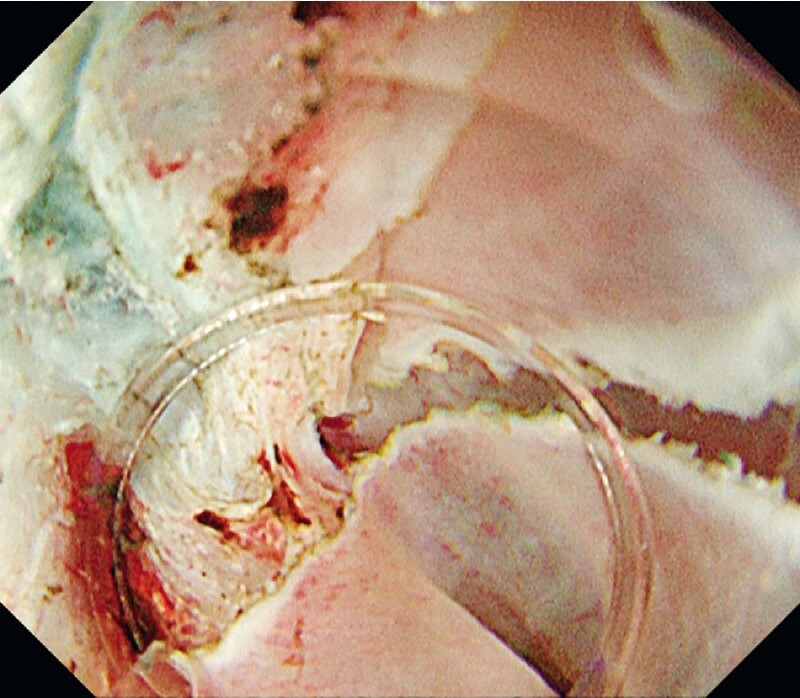
A white light image of a three-dimensional structure with severe fibrosis. We could get into the narrow space with a tapered therapeutic hood and perform precise ESD.

**Fig. 4:**
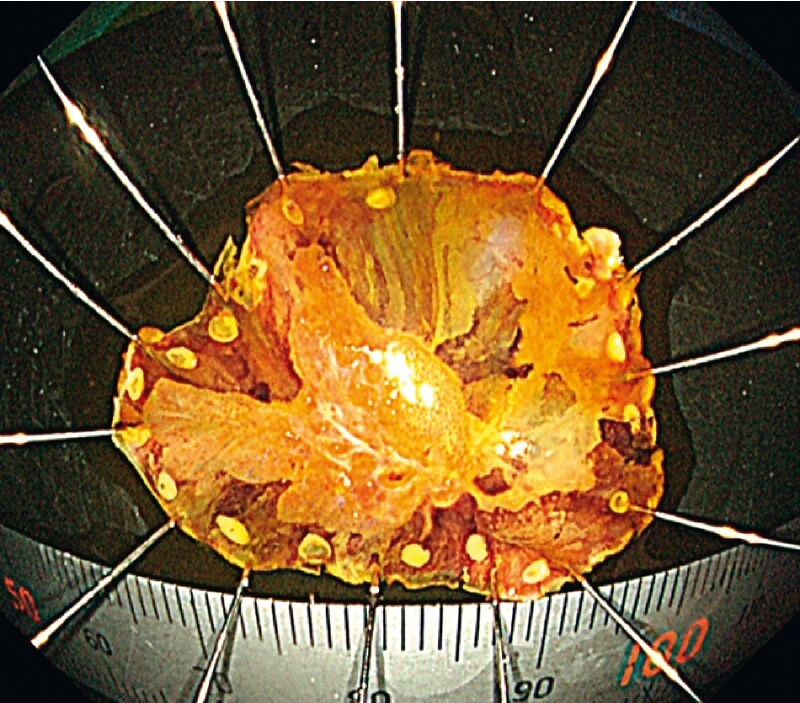
A white light image of specimen with iodine staining. En bloc resection was achieved without any adverse events.

**Fig. 5:**
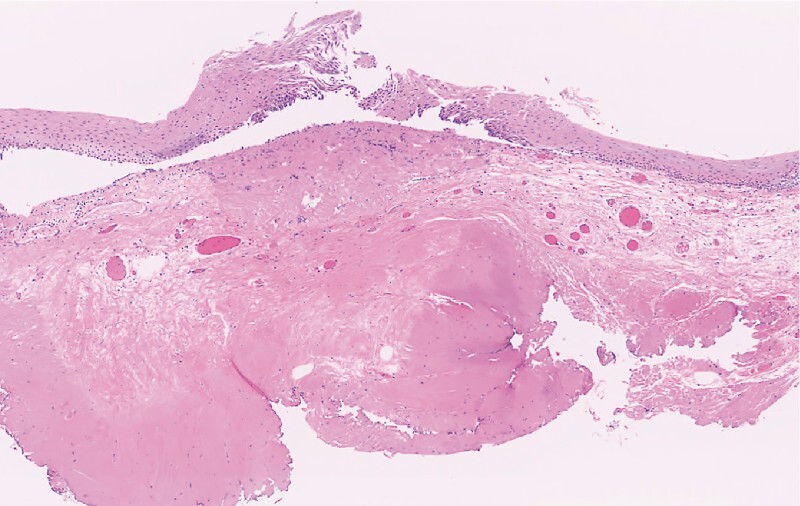
An image of the specimen stained with hematoxylin and eosin. Severely fibrotic tissue is observed adjacent to the lesion.

The water pressure method seems to be particularly useful for hypopharyngeal lesions because it could overcome the difficulty due to the anatomical features of pharynx, which are the narrow space and undulating structure.

Endoscopy_UCTN_Code_TTT_1AQ_2AD

## Notes

Endoscopy E-Videos

https://eref.thieme.de/e-videos
*E-Videos* is an open access online section of the journal *Endoscopy* , reporting on interesting cases and new techniques in gastroenterological endoscopy. All papers include a high-quality video and are published with a Creative Commons CC-BY license. Endoscopy E-Videos qualify for HINARI discounts and waivers and eligibility is automatically checked during the submission process. We grant 100% waivers to articles whose corresponding authors are based in Group A countries and 50% waivers to those who are based in Group B countries as classified by Research4Life (see: https://www.research4life.org/access/eligibility/ ). This section has its own submission website at https://mc.manuscriptcentral.com/e-videos
